# Expression and localization of myosin VI in developing mouse spermatids

**DOI:** 10.1007/s00418-017-1579-z

**Published:** 2017-05-12

**Authors:** Przemysław Zakrzewski, Robert Lenartowski, Maria Jolanta Rędowicz, Kathryn G. Miller, Marta Lenartowska

**Affiliations:** 1 Laboratory of Developmental Biology, Faculty of Biology and Environmental Protection, Nicolaus Copernicus University in Toruń, Toruń, Poland; 2 Laboratory of Isotope and Instrumental Analysis, Faculty of Biology and Environmental Protection, Nicolaus Copernicus University in Toruń, Toruń, Poland; 3 Laboratory of Molecular Basis of Cell Motility, Department of Biochemistry, Nencki Institute of Experimental Biology, Polish Academy of Sciences, Warsaw, Poland; 4 Department of Biology, Washington University in St. Louis, St. Louis, MO USA

**Keywords:** Actin, Immunocytochemistry, Myosin VI splice variants, Spermiogenesis, Ultrastructure

## Abstract

Myosin VI (MVI) is a versatile actin-based motor protein that has been implicated in a variety of different cellular processes, including endo- and exocytic vesicle trafficking, Golgi morphology, and actin structure stabilization. A role for MVI in crucial actin-based processes involved in sperm maturation was demonstrated in *Drosophila*. Because of the prominence and importance of actin structures in mammalian spermiogenesis, we investigated whether MVI was associated with actin-mediated maturation events in mammals. Both immunofluorescence and ultrastructural analyses using immunogold labeling showed that MVI was strongly linked with key structures involved in sperm development and maturation. During the early stage of spermiogenesis, MVI is associated with the Golgi and with coated and uncoated vesicles, which fuse to form the acrosome. Later, as the acrosome spreads to form a cap covering the sperm nucleus, MVI is localized to the acroplaxome, an actin-rich structure that anchors the acrosome to the nucleus. Finally, during the elongation/maturation phase, MVI is associated with the actin-rich structures involved in nuclear shaping: the acroplaxome, manchette, and Sertoli cell actin hoops. Since this is the first report of MVI expression and localization during mouse spermiogenesis and MVI partners in developing sperm have not yet been identified, we discuss some probable roles for MVI in this process. During early stages, MVI is hypothesized to play a role in Golgi morphology and function as well as in actin dynamics regulation important for attachment of developing acrosome to the nuclear envelope. Next, the protein might also play anchoring roles to help generate forces needed for spermatid head elongation. Moreover, association of MVI with actin that accumulates in the Sertoli cell ectoplasmic specialization and other actin structures in surrounding cells suggests additional MVI functions in spermatid movement across the seminiferous epithelium and in sperm release.

## Introduction

**Fig. 1:**
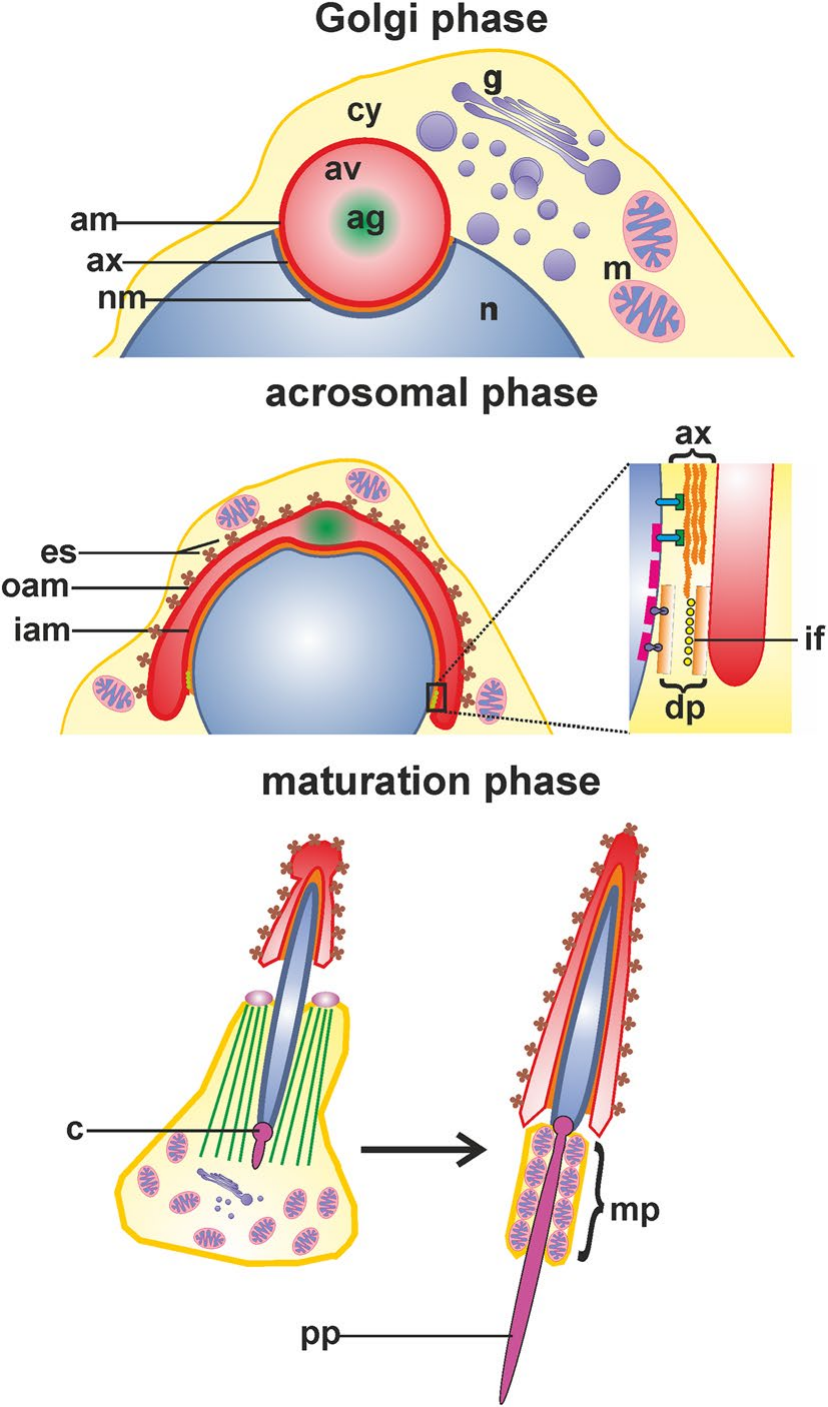
Schematic representation of the main stages of spermiogenesis in mouse: the Golgi, acrosomal, and maturation phases. *ag* acrosomal granule, *am* acrosome membrane, *av* acrosome vesicle, *ax* acroplaxome, *c* centriole, *cy* cytoplasm, *dp* dense plaque, *es* apical ectoplasmic specialization, *g* Golgi apparatus, *iam* inner acrosomal membrane, *if* intermediate filaments, *m* mitochondria, *mp* midpiece, *n* spermatid nucleus, *nm* nuclear envelope, *oam* outer acrosomal membrane, *pp* principal piece

Spermiogenesis is a complex developmental process that entails extensive morphological and biochemical alternations resulting in formation of fully differentiated male gametes—spermatozoa. Two key events of spermiogenesis are acrosome biogenesis and nuclear shaping, accompanied by sperm tail formation. This process in mammals is typically divided into three main phases, during which round spermatids transform into elongated mature sperm (Fig. 1): the Golgi, acrosome cap/elongation, and maturation phases (see review by Toshimori 2009). During the Golgi stage, Golgi-derived proacrosomal vesicles form the acrosome adjacent to the anterior pole of the spermatid nucleus. These granules tether, dock, and fuse along the acroplaxome, a cytoskeletal plate stabilized by the keratin 5/Sak57-containing marginal ring that anchors the developing acrosome to the nuclear envelope (Kierszenbaum et al. 2003b, 2011 and see review by Kierszenbaum and Tres 2004). The pair of centrioles migrates distally and initiates formation of the flagellum. A cloud-like structure, called the chromatoid body, establishes contact with intranuclear material through the pore complexes at the caudal pole of the spermatid nucleus. Next, the giant acrosomal vesicle spreads over the spermatid nucleus to form a distinct cap, while the Golgi complex migrates toward the posterior pole of the sperm nucleus. Soon after acrosome biogenesis starts, a transient cytoskeletal structure—the manchette—develops caudally to the acrosome–acroplaxome around the nucleus and the spermatid initiates elongation, starting the acrosome/elongation subphase. Golgi-derived non-acrosomal vesicles are mobilized and transported along the manchette to the centrosome region and developing flagellum (see reviews by Kierszenbaum 2002; Kierszenbaum and Tres 2004). As the elongation progresses, the acrosome contents gradually condense, the cap continues to cover the spermatid nucleus, and distal centriole produces an axoneme. During the last step of spermiogenesis, the spermatid nucleus is remodeled by chromatin condensation, the manchette disappears upon completion of the sperm head elongation, and mitochondria are packed into the midpiece of elongating tail (see review by Toshimori 2009). Immediately prior to spermiation, excess cytoplasm is eliminated from the future sperm as the residual body, which is phagocytosed by the surrounding Sertoli cell.

The actin cytoskeleton, including a number of actin-binding/regulating proteins (ABPs), has been implicated in various aspects of mammalian spermiogenesis. First, filamentous actin (F-actin) has been identified as a central component of several unique cytoskeletal structures assembled during spermatid differentiation including the acrosome–acroplaxome complex, the manchette, and the apical ectoplasmic specialization of the Sertoli cell adjacent to developing spermatid (Kierszenbaum et al. 2003b and see reviews by Kierszenbaum et al. 2011; Sun et al. 2011; Qian et al. 2014). Second, two different molecular motor systems operate to mobilize vesicle cargos required for acrosome biogenesis and tail development. Besides microtubules (see reviews by Berruti and Paiardi 2011; Lehti and Sironen 2016), an actin-related pathway using the MVa/Rab27a/b complex is involved in Golgi-derived vesicle transport along the acroplaxome and the manchette (Kierszenbaum et al. 2003a, 2004; Hayasaka et al. 2008). In addition, MVa-decorated vesicles surround a portion of the chromatoid body, suggesting a possible role of actin filaments in the disposal of nuclear material generated during spermiogenesis (Kierszebaum et al. 2003a and see review by Kierszenbaum and Tres 2004). Third, the acrosome–acroplaxome–manchette complex contains ABPs such as cortactin and profilin-3, which are thought to modulate actin dynamics during acrosomogenesis and head shaping (Obermann et al. 2005; Kierszenbaum et al. 2008; Behnen et al. 2009). Finally, the apical ectoplasmic specialization associated with the tubulobulbar complexes at the concave side of the elongating spermatid head contains actin filaments. These actin structures form a stack of hoops stabilized by espin and adhesion protein complexes (Kierszenbaum et al. 2003b and see reviews by Kierszenbaum and Tres 2004; Kierszenbaum et al. 2007; Xiao and Yang 2007). Spatiotemporal expression of testis-specific actin assembly/disassembly regulators modulates adhesion of spermatids to the Sertoli cells during their movement across the seminiferous epithelium, and then allows the release of mature sperm at spermiation. Although F-actin structures seem to play important roles during the key events of spermiogenesis in mammals, the molecular basis of their regulation and roles in the processes is still poorly understood.

During *Drosophila* spermatogenesis, some processes similar to those described for mammalian spermatogenesis occur, and the actin cytoskeleton plays several important roles. Stable actin structures, called actin cones, mediate spermatid individualization during the final step of *Drosophila* spermiogenesis when 64 syncytial spermatids are reorganized into individual mature sperms (Noguchi and Miller 2003; Noguchi et al. 2006). As these cones move along the axonemes from the spermatid nuclei to the end of the tails, cytoplasm is removed from maturing spermatids and the cyst membrane is remodeled into individual sperm membranes. Actin cones are composed of two structural domains, a front meshwork that excludes the cytoplasmic contents and a tail of parallel bundles driving the cone movement (Noguchi et al. 2006, 2008). We have previously found that localization of MVI to the cones’ fronts is required for their proper formation and function during spermatid individualization (Noguchi et al. 2006; Isaji et al. 2011; Lenartowska et al. 2012). In MVI mutants, actin cone organization is disrupted, leading to cessation of the individualization process and male infertility. In addition, when MVI is absent or mislocalized, distribution of other ABPs is abnormal. Some components usually localized to the front of cones are spread throughout the cones, suggesting that MVI might function by anchoring specific cargos in the front meshwork (Rogat and Miller 2002; Noguchi et al. 2008; Isaji et al. 2011).

MVI is the only known pointed-end-directed actin-based motor (see review by Buss and Kendrick-Jones 2008). Similar to other myosins, MVI has an N-terminal motor domain (containing an ATP-binding pocket and actin-binding interface), a neck or “lever arm” region (binding two calmodulin or calmodulin-like light chains), and a tail with the C-terminal cargo-binding domain. MVI also contains a two unique inserts in the head/neck region, including a 22-aa Insert2, responsible for minus end-directed movement along actin. Moreover, four alternative MVI splice variants have been identified in mammals, containing a large insert, a small insert, both inserts, or no insert within the C-terminal tail. These isoforms are differentially expressed in different tissues/cell types and are associated with specific subcellular compartments and functions. MVI has been implicated in several processes through functional studies in flies, worms, and mammals, including clathrin-mediated endocytosis, Golgi organization and secretion, basolateral targeting and sorting, cell adhesion and epithelial integrity, cell migration, actin dynamics, cytokinesis, transcription (see review by Buss and Kendrick-Jones 2008), and myogenesis (Karolczak et al. 2013, 2015). In these seemingly different cellular processes, MVI may function as a cargo transporter or as a protein anchor involved in actin organization/dynamics in specialized cells.

Mutation in the MVI gene in *Snell’s waltzer* mice (*sv/sv* mutants) leads to deafness as a result of neurosensory epithelia degeneration in the inner ear (Avraham et al. 1995; Self et al. 1999). These mice display also several other defects in different cell types such as aberrations in Golgi morphology, reduced secretion, defective endocytosis, and impaired morphology of brush border enterocytes and hippocampal neurons. In addition, profound fibrosis and both cardiac and pulmonary vascular endothelial defects were also observed (Hegan et al. 2012, 2015 and references therein). Although *sv/sv* males exhibit somewhat reduced fertility (Avraham et al. 1995 and our unpublished observations), no studies have been published that address the possible role of MVI in mouse spermatogenesis. However, given that: (1) dynamic actin structures modulated by specific ABPs determine the success of spermiogenesis in both invertebrate and mammals, and (2) MVI is a key element of these functional actin-related protein complexes during spermatid maturation in *Drosophila*, we hypothesized that MVI may be involved in mammalian spermatid maturation. To test this hypothesis, we examined the MVI expression and localization using immunocytochemical approaches complemented with ultrastructural analysis of mouse testes. To the best of our knowledge, this is the first detailed study of MVI during spermatid development in mammals.

## Materials and methods

### Animals

Wild-type adult male mice were used in the study. All animal work, until the mouse tissues were harvested, was performed at the Nencki Institute of Experimental Biology (Warsaw, Poland). Animal housing and killing procedures were performed in compliance with the European Communities Council directives adopted by the Polish Parliament (Act of 15 January 2015 on the use of animals in scientific investigations). All conducted experiments were repeated a minimum of three times with similar results.

### MVI splice variant analysis by RT-PCR

To determine the MVI isoform(s) expressed in mouse testes, organs were dissected from adult males and total RNA was extracted with TRI Reagent^®^ (Sigma-Aldrich) according to the manufacturer’s protocol. First-strand cDNA synthesis was performed with 1 μg of total RNA, dART reverse transcriptase, and an oligo(dT)_20_ primer following the manufacturer’s instruction (EURx). Nested PCR was done to identify the splicing isoforms of the mouse MVI. A 2 μl of first-strand cDNA was used as template for PCR amplification with OptiTaq DNA polymerase and outer gene-specific primers (forward 5′-GATGAGGCACAGGGTGAC-3′ and reverse 5′-TTGTTCTGAGGGTCTTTGTA-3′). A 2-μl aliquot of the first PCR mixture served as the template in a second PCR using the inner gene-specific primers (forward 5′-ATGAGGCACAGGGTGACAT-3′ and reverse 5′-TTCTGAGGGTCTTTGTACTGGT-3′). PCR cycles were as follows: 95 °C for 2 min followed by 35 cycles of 95 °C for 30 s, 57 °C for 30 s, 72 °C for 30 s, followed by a final extension step of 72 °C for 10 min. The PCR products were visually inspected on a 2% agarose gel in TBE buffer.

### Immunoblotting

To verify the specificity of the commercial primary antibodies in mouse used during the subsequent immunolocalization studies, testes, liver, kidneys, heart, lungs, and brain dissected from the male adult mice were homogenized in liquid nitrogen, and soluble proteins were extracted in 100 mM Tris–HCl (pH 7.5), 5 mM EDTA, 5 mM EGTA, 10% sucrose, and Complete Protease Inhibitor Cocktail (Roche) according to the manufacturer’s protocol. The homogenates were centrifuged at 16,000*g* for 30 min at 4°C and concentrations of the supernatants were measured with the Bio-Rad DC Protein Assay according to the manufacturer’s instructions. Equal amounts of proteins were separated by electrophoresis on a 7.5% SDS-PAGE gels and then the proteins were semi-dry transferred to Amersham PVDF Hybond-P membrane (GE Healthcare). Blocked blots were probed with a rabbit polyclonal antibody against MVI at 1:50 dilution (MVI PAb, Proteus) or a mouse monoclonal anti-actin antibody (JLA20 MAb, Calbiochem) at 1:5000 dilution, washed, and probed with the corresponding anti-rabbit IgG or anti-mouse IgG/IgM secondary antibodies, conjugated with horseradish peroxidase (HRP, Sigma-Aldrich and Merck, respectively). Signals were detected with the Amersham ECL Advance Western Blotting Detection Kit according to the manufacturer’s guidelines (GE Healthcare).

### Immunofluorescence studies

Dissected testes were fixed with 4% (v/v) formaldehyde and 0.25% (v/v) glutaraldehyde in 0.1 M phosphate-buffered saline (PBS, pH 7.4) for 2 h at room temperature (slight vacuum infiltration). Pre-fixed testes were then cut into small pieces and further fixation was proceeded overnight at 4 °C. Fixed samples were washed with PBS, dehydrated in a graded series of increasing ethanol concentrations, and embedded in LR Gold resin (Sigma-Aldrich) according to the standard protocol. Samples were then sectioned with a diamond knife into semithin sections (cross sections of seminiferous tubules) and transferred onto microscope slides covered with Biobond (BB International). For preliminary analysis, sections were stained with 0.1% toluidine blue according to the standard protocol and observed under the light microscope. For immunolocalization (single labeling technique), sections were blocked with 1% (MVI localization) or 3% (actin localization) bovine serum albumin (BSA, Sigma-Aldrich) for 2 h and then incubated with the primary MVI PAb or the JLA20 MAb overnight at 4 °C, at dilutions 1:50 or 1:500, respectively. Signals were detected using the corresponding anti-rabbit IgG Cy3^®^ (Sigma-Aldrich) or anti-mouse IgG/IgM Alexa Fluor 488^®^ secondary antibodies (ThermoFisher). In the final step, DNA was stained with 2 μg/ml 4′,6-diamidino-2-phenylindole (DAPI, Fluka). Specimens were covered with MobiGLOW mounting medium (MoBiTec) to prolong the fluorescence. Negative controls were processed in the same way except that no primary antibodies were added. Images were acquired using an Olympus BX50 fluorescence microscope, Olympus Xc50 digital color camera, and cellB software (Olympus Soft Imaging Solutions gmbH).

### Immunogold electron microscopy

For detailed ultrastructural analysis, dissected testes were fixed in 2% (v/v) glutaraldehyde in 0.1 M PBS (pH 7.4) for 2 h at room temperature (slight vacuum infiltration). Pre-fixed testes were then cut into small pieces and further fixation was proceeded overnight at 4 °C. Next, the samples were post-fixed with 1% (v/v) osmium tetroxide (Polysciences) in PBS for 2 h at 4 °C, dehydrated in ethanol, and embedded in Spurr resin (Sigma-Aldrich) according to the standard protocol. Ultrathin sections (cross sections of seminiferous tubules) were collected on copper grids, post-stained with 5% uranyl acetate and 0.4% lead citrate solutions, and examined on a Joel EM 1010 transmission electron microscope.

For post-embedding immunogold MVI localization in developing spermatids, samples were prepared according to the same protocol as for previous immunolocalization. Ultrathin cross sections of seminiferous tubules were cut, collected on nickel grids, and incubated with blocking solution containing 1% BSA in 0.1 M PBS (pH 7.4) for 5 min at room temperature. Next, sections were incubated in 1:100 dilution of the MVI PAb in PBS supplemented with 0.1% BSA for 1.5 h, followed by incubation with a gold-conjugated anti-rabbit IgG 15-nm secondary antibody (BB International) at dilution 1:100 in PBS with 0.1% BSA for 45 min. Both incubations were proceeded at room temperature. In the negative control, the primary antibody was omitted. Finally, the sections were post-stained with 2.5% uranyl acetate and 0.4% lead citrate solutions and examined by transmission electron microscopy as above.

## Results

### MVI expression in mouse testis

**Fig. 2:**
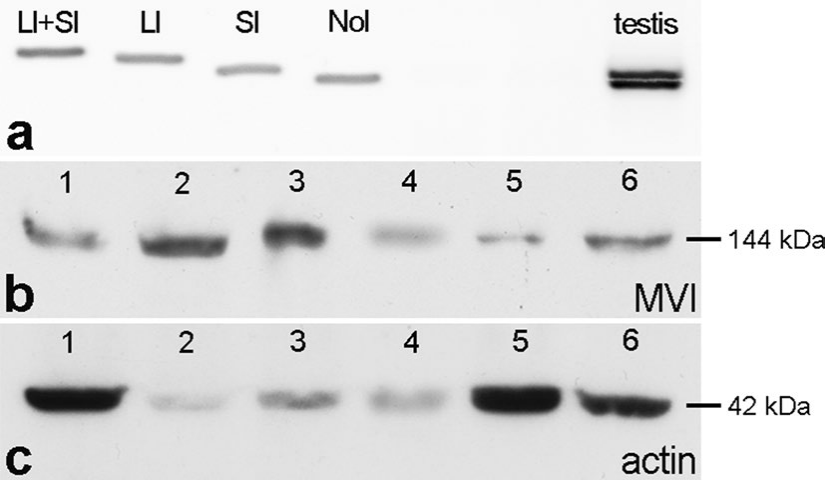
Verification of MVI splice variants expressed in mouse testes and specificity of used commercial antibodies in these organs.** a** RT-PCR products obtained with primers designed to produce MVI fragments containing either a large insert (LI), a small insert (SI), both inserts (LI + SI) or no insert (NoI) from control plasmids (*first four lanes*) and mouse testis (*last lane*).** b**,** c** Immunoblotting of crude protein extracts from different mouse tissues with MVI PAb (**b**) and anti-actin JL20 MAb antibodies (**c**). *Lane 1* testis, *2* liver, *3* kidney, *4* heart, *5* lung, *6* brain

Four MVI posttranscriptional splice variants (Fig. 2a) can be expressed in mammals due to the presence of two inserts [long (LI) and short (SI)] in the C-terminal globular tail: MVI with LI only, with SI only, with both long and short (LI + SI) or with no insert (NoI). Given that these isoforms are differentially expressed in various tissues/cell types where they have diverse localization and function, we first examined which of the MVI splice variants were expressed in mouse testes. To establish this, RT-PCR was performed. Bands corresponding to SI and NoI MVI tail isoforms were detected. Thus, these two isoforms are the primary isoforms expressed in mouse testis (Fig. 2a, last lane).

We next performed immunofluorescence studies of MVI and actin distributions during mouse spermatogenesis. Because we used commercial primary antibodies, western blot analysis was performed to confirm the specificity of MVI PAb and JLA20 MAb in mouse testes (Fig. 2b, c). Our western blots showed that both antibodies recognized the appropriately sized target proteins in different mouse tissues, including testes (Fig. 2b, c, lane 1).

**Fig. 3:**
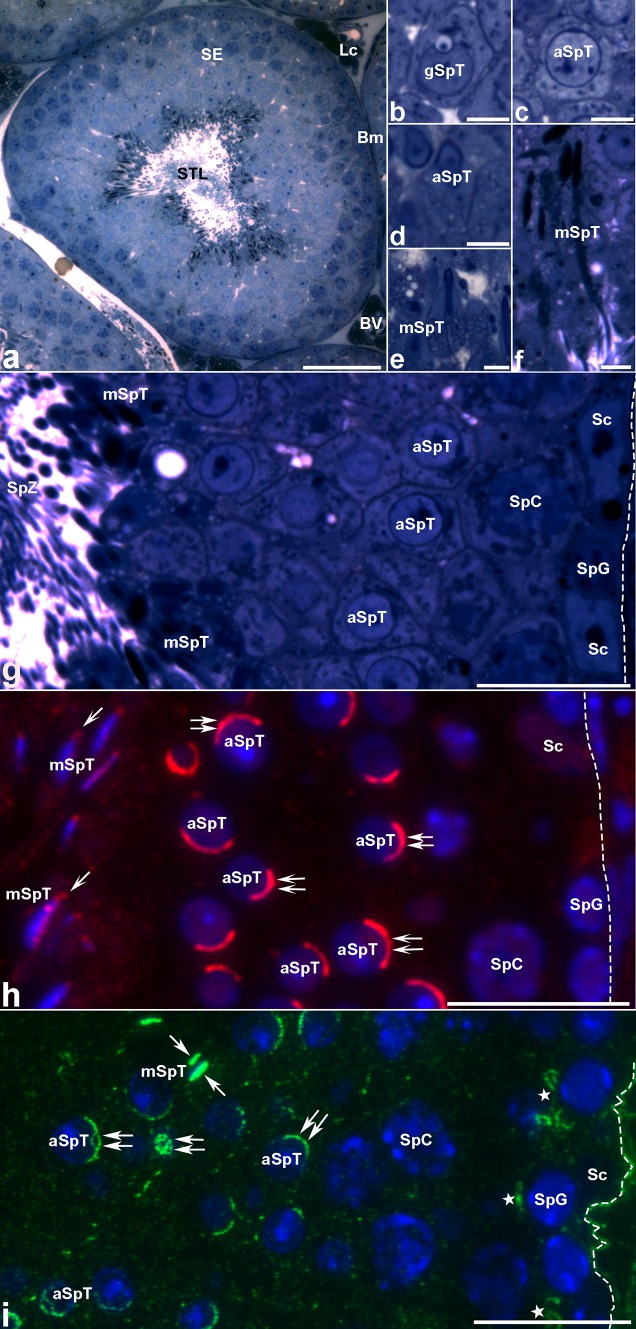
Toluidine blue staining (**a**–**g**) and immunofluorescence labeling of MVI (**h**) and actin (**i**) of mouse seminiferous tubules during spermatogenis. *aSpT* spermatid at the acrosome phase, *BV* blood vessel, *gSpT* spermatid at the Golgi phase, *Lc* Leydig cell, *mSpT* spermatid at the maturation phase, *Sc* Sertoli cell, *SE* seminiferous epithelium, *SpC* spermatocyte, *SpG* spermatogonium, *SpZ* spermatozoa, *STL* seminiferous tubule lumen. *Arrows* or *double arrows* show MVI (*red*) and actin (*green*) staining in spermatids at maturation or acrosome phase, respectively; *stars* in** i** show actin localization in basal ectoplasmic specialization; *dashed lines* basement membrane. Nuclei are stained with DAPI (*blue*). *Bars* 50 μm (**a**), 20 μm (**g**–**i**), 5 μm (**b-f**)

**Fig. 4:**
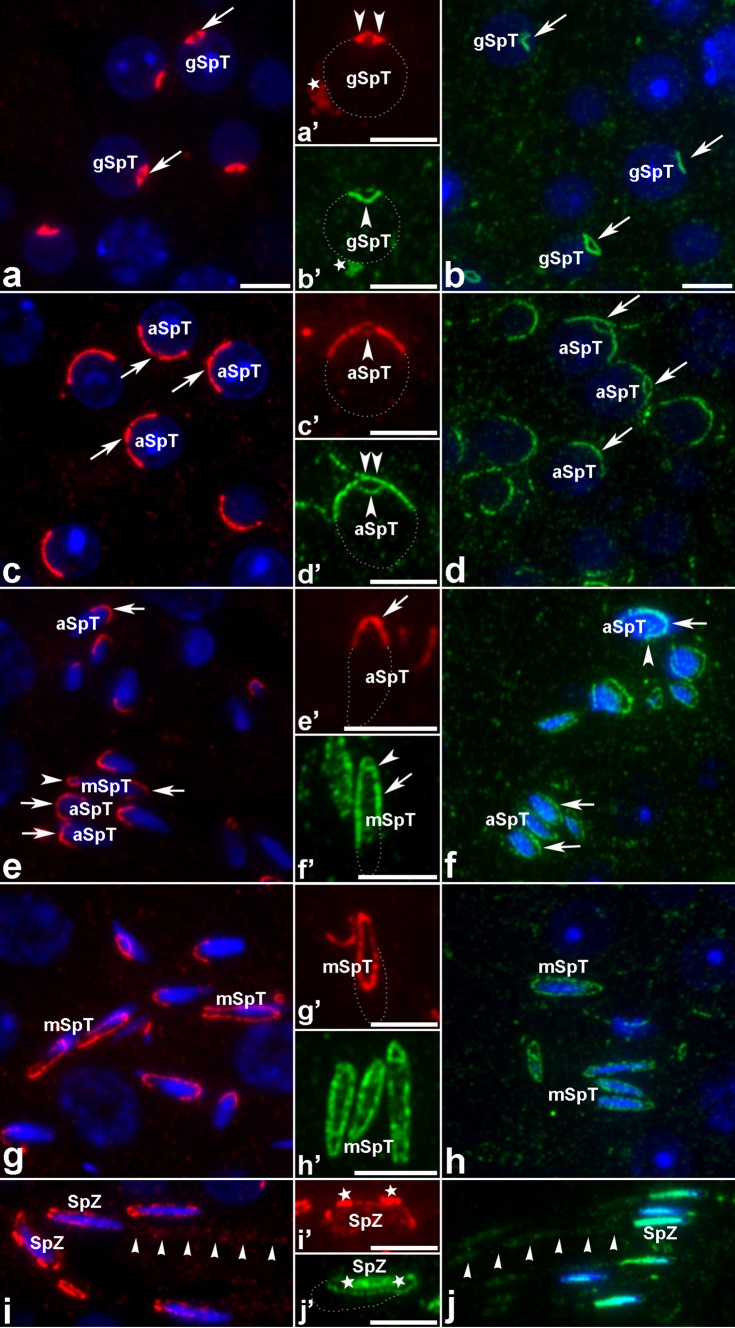
Immunofluorescence localization of MVI (*red*) and actin (*green*) in mouse developing spermatids during the Golgi phase (gSpT), the acrosomal phase (aSpT), and the maturation phase (mSpT) as well as in spermatozoa (SpZ). All other *indications* are explained in the text (see “Results”). Nuclei are stained with DAPI (*blue*) or outlined with *dashed line*. *Bars* 5 μm

Immunofluorescence localizations of MVI (Figs. 3, 4, red) and actin (Figs. 3, 4, green) were performed to examine distributions of these proteins in the seminiferous epithelium. As shown in the toluidine-stained semithin cross sections, seminiferous tubules contain differentiating generative line cells including spermatogonia, spermatocytes, and spermatids, associated with somatic Sertoli cells (Fig. 3a–g). Successive developmental phases were visible, including the Golgi phase (Fig. 3b), the acrosome/cap phase (Fig. 3c), the acrosome/elongation phase (Fig. 3d), and finally the maturation phase (Fig. 3e, f). MVI was preferentially localized to the acrosomes that spread over the spermatid nuclei at the acrosome stage (Fig. 3h, double arrows) and to the elongated spermatid heads (Fig. 3h, arrows). In the developing spermatid acrosomes during the acrosomal and maturation phases, MVI and actin are both present (compare Fig. 3h, i, double arrows and arrows), suggesting that MVI is associated with actin-based processes involved in sperm development and maturation. Actin staining was also found within the cytoplasm of the seminiferous epithelium cells (Fig. 3i) and accumulated in the basal ectoplasmic specializations (Fig. 3i, stars) and the basement membrane of the testis (Fig. 3i, dotted line). No labeling was observed when the primary antibodies were omitted (data not shown).

Because the most intensive MVI PAb and JLA20 MAb immunoreactivities were observed in developing spermatids during the transformation into mature spermatozoa, further detailed analysis of MVI and actin distributions was performed during this prolonged cell differentiation stage. Observations focused on the Golgi phase, the acrosome cap/elongation phase, and the maturation phase (Fig. 4). During the early stage of the acrosome biogenesis, the strongest immunofluorescence signals for MVI and actin were associated with the nascent acrosome vesicle (Fig. 4a, b, arrows). However, localization patterns of these two proteins in developing acrosome were somewhat different. While MVI was found in the acrosomal sac with the exception of the hydrolytic enzyme-rich interior (Fig. 4a′, arrowheads), actin staining was strictly limited to the acrosome–acroplaxome complex linking developing acrosome with the spermatid nucleus (Fig. 4b′, arrowhead). Both proteins were also localized in some regular spots detectable within the cytoplasm of round spermatids and adjacent to the spermatid nuclei (Fig. 4a′, b′, stars). These MVI/actin-enriched spots may correspond with the chromatoid bodies. When the giant acrosome vesicle spread over the nucleus, MVI and actin were associated with the acrosome (Fig. 4c, d, arrows). However, the actin labeling was distinctly stronger in the acrosome–acroplaxome complex and at the top of the acrosome vesicle (compare Fig. 4c′, d′, arrowheads, and double arrowhead). Similar patterns of MVI and actin distributions were observed during the acrosome/elongation and early/late maturation phases (Fig. 4e–h). When the spermatid nuclei started elongation, MVI was detectable mainly at the tip region of the acrosome (Fig. 4e, e′, arrows) while actin was clearly visible along the acrosome–acroplaxome complex (Fig. 4f,f' arrows), including the top of the acrosome vesicle (Fig. 4f', arrowhead) and the acroplaxome marginal ring region (Figs. 4f, arrowhead). As spermatid elongation progressed, both proteins were distributed along the acrosome–acroplaxome complex (Fig. 4e–h), including the post-acrosomal region (Fig. 4e, arrowhead, and g–h). In addition, actin staining was detected inside the nuclei during spermatid elongation and maturation (Fig. 4f, f′, h, h′). Finally, just before spermiation, MVI and actin were accumulated around the elongated sperm heads (Fig. 4i, j), particularly in the regions adjacent to the apical ectoplasmic specializations (Fig. 4i′, j′, stars). MVI was also detected inside the sperm nuclei (Fig. 4i′) and in the midpiece of the sperm tail (Fig. 4i, arrowheads) along with actin (Fig. 4j, sperm nuclei and mid piece pointed by arrowheads).

### Ultrastructural analysis of MVI localization in mouse spermatids

We next sought to determine distribution of MVI in developing spermatids at ultrastructural level using immuno-electron microscopy with the MVI PAb and a gold-conjugated secondary antibody. Since the fixation for immunocytochemistry usually does not preserve some organelles and cell structures/membranes well enough for ultrastructural localization, our immunogold experiments were completed using conventionally fixed ultrathin sections. With this combined strategy, we were able to distinguish MVI localization in defined sub-domains of cell compartments (such as the *trans*-Golgi network, TGN) or highly specialized cell structures (such as the acroplaxome).

### Golgi phase

**Fig. 5:**
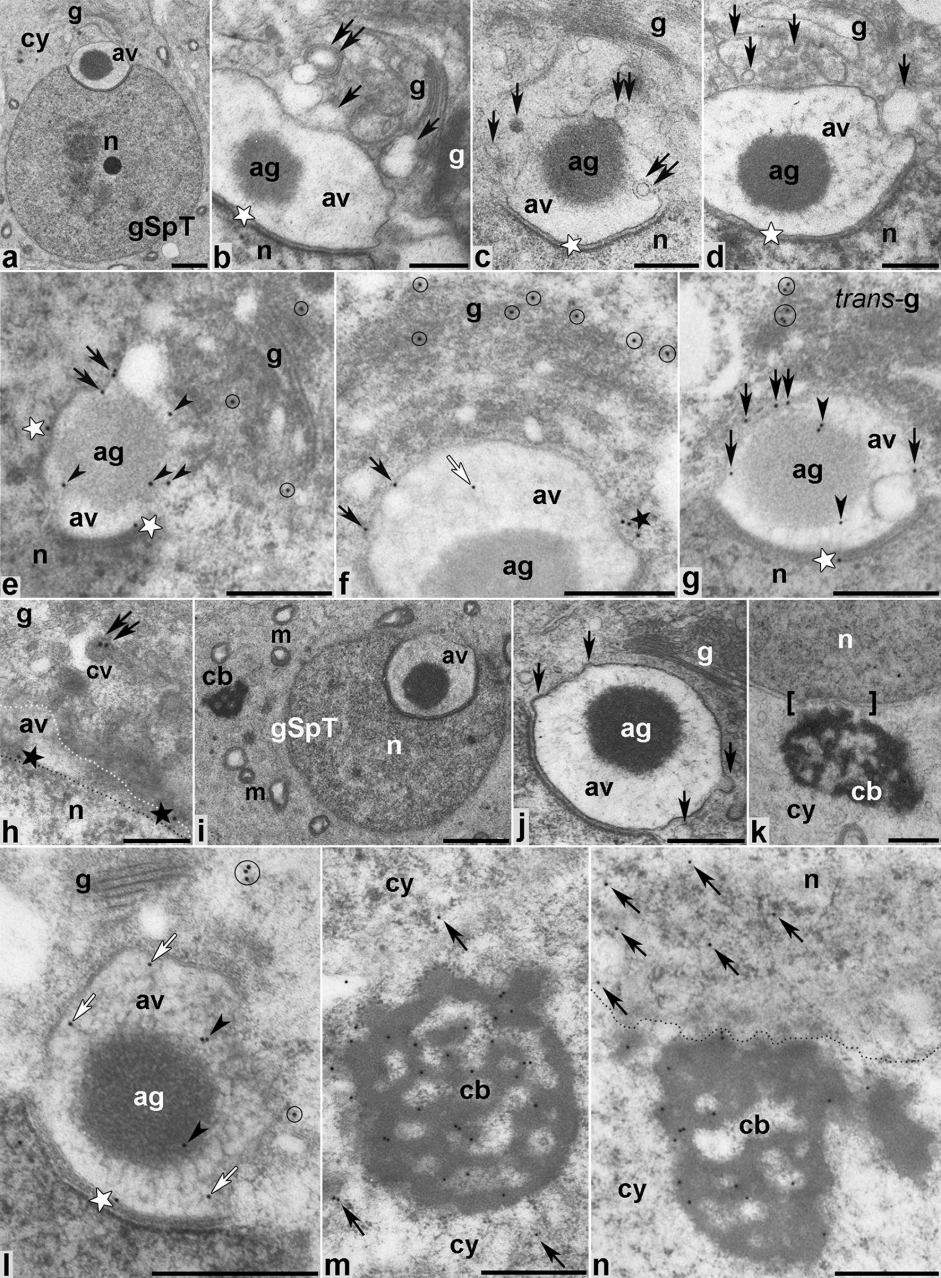
Ultrastructural analysis (**a**–**d**,** i**–**k**) and immunogold localization (**e**–**h**,** l**–**n**) of MVI in developing mouse spermatids during the Golgi phase. *ag* acrosomal granule, *av* acrosome vesicle, *cb* chromatoid body, *cy* cytoplasm, *g* Golgi complex, *gSpT* spermatid at the Golgi phase, *m* mitochondria, *n* nucleus. *Square brackets* in k show the contact region between the nucleus and the chromatoid body. *Dotted lines* mark a boundary between the spermatid cytoplasm, the acrosomal vesicle and the nucleus (h) or between the spermatid cytoplasm and the nucleus (n). All other *indications* are explained in the text (see “Results”). *Bars* 1 μm (**a**,** i**), 500 nm (**b**–**g**,** i**–**n**), 250 nm (**h**)

During the Golgi phase, the giant acrosomal vesicle, which contains electron-dense acrosomal granule material, is formed by numerous Golgi-derived vesicles (Fig. 5a–d), including both uncoated (Fig. 5b–d, arrows) and clathrin-coated (5b, c, double arrows) vesicles. These vesicles dock and fuse along the actin-containing plaque acroplaxome that links developing acrosome to the spermatid nuclear envelope (Fig. 5b–d, stars). As shown in Fig. 5e–g, gold particles representing MVI location were observed in the Golgi region adjacent to the acrosome-nuclear pole of round spermatids, including the TGN enriched with proacrosomal vesicles (Fig. 5e–g, circles). Immunogold labeling showed MVI localization along the outer acrosome membrane (Fig. 5e–g, arrows) and in the inner acrosome membrane–acroplaxome interface (Fig. 5e, g, stars), including developing acrosome–acroplaxome leading edges (Fig. 5f, h, stars). Some gold traces were also found on the surface of the acrosomal granule (Fig. 5e, g, arrowheads) and occasionally within the acrosome vesicle space (Fig. 5f, white arrow). We also detected MVI associated with clathrin-coated vesicles near the acrosomal outer membrane (Fig. 5h, double arrow). During the Golgi phase, the electron-dense granulo-filamentous chromatoid body was clearly visible in the spermatid cytoplasm (Fig. 5i). As acrosome biogenesis progressed and Golgi-derived vesicles continued to fuse with developing acrosome (Fig. 5j, arrows), the chromatoid body migrated to the caudal cytoplasmic region of the round spermatid and established contact with the nuclear envelope (Fig. 5k, square brackets). At this stage, MVI was still apparent in the spermatid cytoplasm around the acrosome (Fig. 5l, circles) and in the acroplaxome (Fig. 5l, star). However, some gold traces were also detected inside the acrosome vesicle; they were localized near the acrosome membrane (Fig. 5l, arrows) or on the periphery of the acrosomal granule (Fig. 5l, arrowheads). In addition, a strong MVI immunoreactivity was associated with electron-dense fibrillar material of the chromatoid body near the spermatid nucleus, as well as adjacent to the spermatid nucleus (Fig. 5m, n, respectively). Numerous gold traces were also found within the chromatin (Fig. 5n, arrows), while only few were localized in the posterior region of the spermatid cytoplasm (Fig. 5m, arrows).

### Acrosome cap/elongation phase

**Fig. 6:**
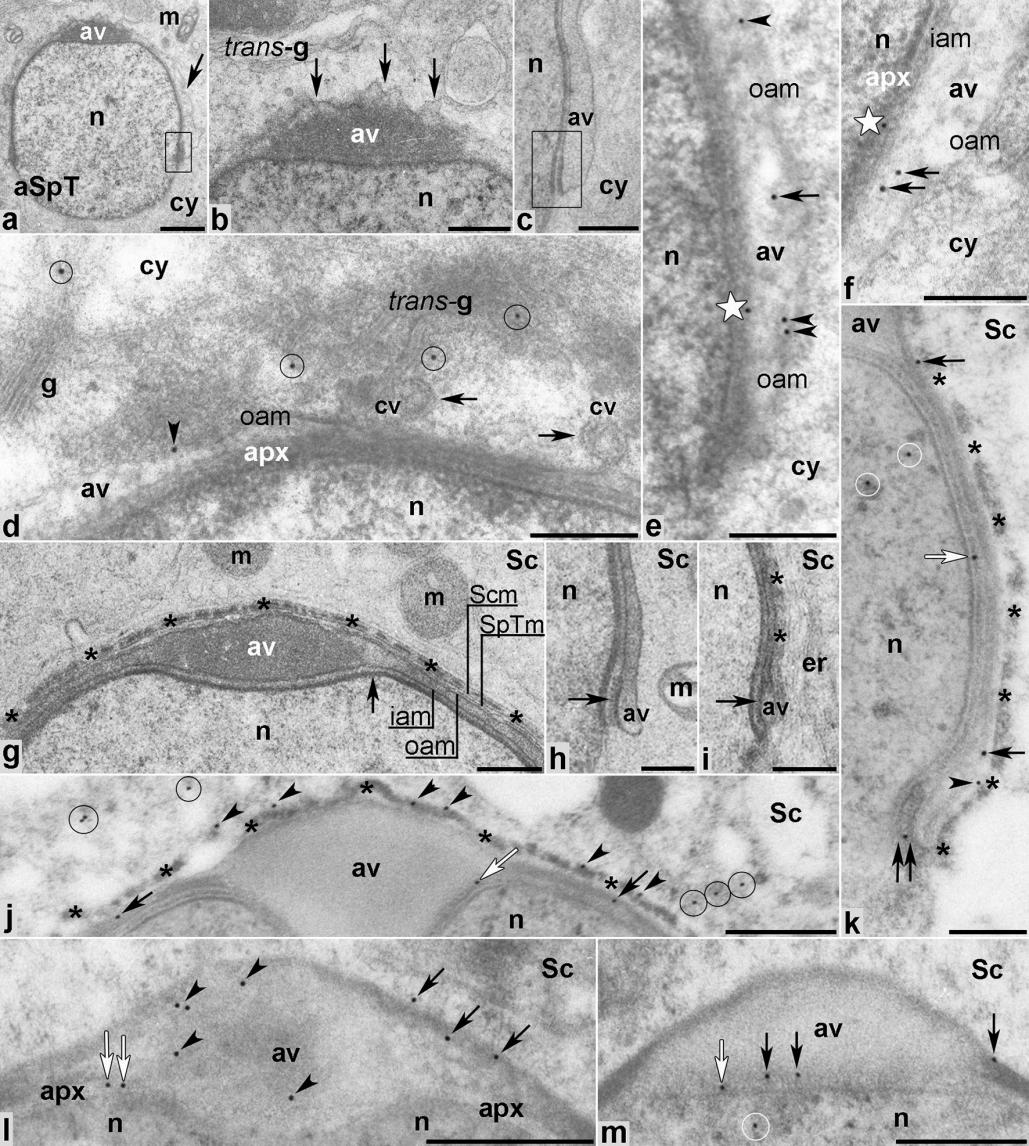
Ultrastructural analysis (**a**–**c**,** g**–**i**) and immunogold localization (**d**–**f**,** j**–**m**) of MVI in developing mouse spermatids during the acrosome phase. *apx* acroplaxome, *aSpT* spermatid at the acrosome phase, *av* acrosome vesicle, *cy* cytoplasm, *er* endoplasmic reticulum, *g* Golgi complex, *iam* inner acrosome membrane, *m* mitochondria, *n* nucleus, *oam* outer acrosome membrane, *Sc* Sertoli cell, *Scm* Sertoli cell membrane, *SpTm* spermatid membrane. *Boxed regions* in** a** and** c** include the acrosome–acroplaxome marginal ring regions. All other *indications* are explained in the text (see “Results”). *Bars* 1 μm (**a**), 500 nm (**b**,** d**,** j**–**l**), 250 nm (**g**–**i**,** m**), 200 nm (**c**,** e**,** f**)

During the acrosome phase, the acrosome vesicle spreads over the spermatid nucleus to form a distinct cap and the spermatid initiates its elongation (Fig. 6). At the early cap subphase, the outer acrosomal membrane was strongly pleated, demonstrating that Golgi-derived vesicles still undergo fusion during this period (Fig. 6a, b, arrows). We could also discern the inner acrosomal membrane-associated plaque at the leading edge of the acrosome–acroplaxome complex (Fig. 6a, c, boxed regions). As before, MVI was present in the TGN (Fig. 6d, circles), enriched with the clathrin-coated vesicles that undergo fusion with the acrosome vesicle (Fig. 6d, arrows). MVI was also localized in the outer acrosomal membrane (Fig. 6d, e arrowheads). In the acroplaxome marginal ring zone, gold traces were found in the interspace between the outer and inner acrosomal membranes (Fig. 6e, f arrows) and in the acroplaxome (Fig. 6e, f stars).

As the acrosome phase progressed, distinct structural elements of the spermatid anterior pole were distinguishable (Fig. 6g), such as the spermatid membrane, the acrosome cap, the acrosome outer and inner membranes, and the acroplaxome (Fig. 6g, arrow). At higher magnification, we could also discern the acrosomal electron-dense plaque in the acroplaxome marginal ring (Fig. 6h, i, arrows). The Sertoli cell membrane (Fig. 6g) together with adjacent ectoplasmic F-actin bundles (Fig. 6g, i, asterisks) and the endoplasmic reticulum cisternae (Fig. 6i, *er* ) were also clearly visible along the spreading acrosome. During this subphase, a pattern similar to the previously described MVI localization was observed, with immunogold staining present in the outer/inner acrosome membranes (Fig. 6j–m, black arrows) and in the acroplaxome (Fig. 6j–m, white arrows), including its marginal ring (Fig. 6k, double arrow). We also found MVI in the F-actin-containing apical ectoplasmic specializations (Fig. 6j, k arrowheads) and the Sertoli cell cytoplasm (Fig. 6j, circles). Interestingly, association of MVI with actin filaments in the apical ectoplasmic specializations was not always apparent (compare Fig. 6j, k). This association became more intense as the spermatid matured (see the next results). Gold traces were still present within the spermatid chromatin (Fig. 6k, m, circles).

### Maturation phase

**Fig. 7:**
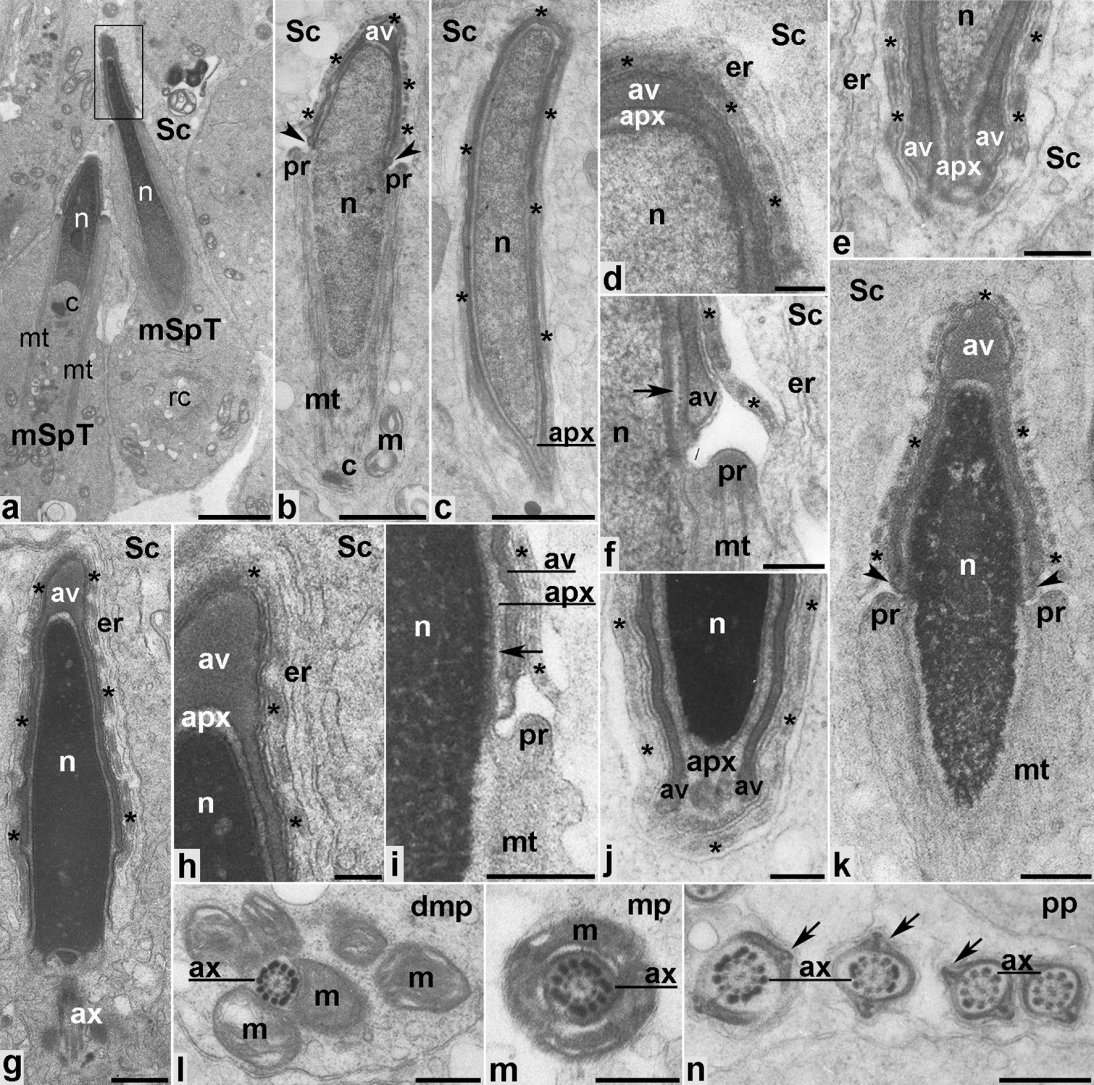
Ultrastructural analysis of developing mouse spermatids during the maturation phase. *apx* acroplaxome, *av* acrosome vesicle, *ax* axoneme, *c* centriole, *dmp* developing midpiece of the sperm tail, *er* endoplasmic reticulum, *m* mitochondria, *mp* midpiece of the sperm tail, *mSpT* spermatid at the maturation phase, *mt* manchette, *n* nucleus, *pp* principal piece of the sperm tail, pr perinuclear ring of the manchette, *Sc* Sertoli cell. *Boxed region* in a includes the spermatid head with spreading acrosome. All other *indications* are explained in the text (see “Results”). *Bars* 2 μm (**a**), 1 μm (**b**,** c**), 500 nm (**g**–**h**,** l**–**n**), 200 nm (**d**–**f**,** i**–**k**)

During the maturation phase, nuclear shaping based on cooperative exogenous and endogenous clutching forces of the Sertoli cell apical ectoplasmic specializations and the acrosome–acroplaxome–manchette complex coincides with spermatid chromatin condensation, sperm tail formation, and residual cytoplasm removal from the future sperm (Fig. 7a). The apical pole of the elongating and condensing spermatid nucleus (Fig. 7a–k) is capped by the homogenous acrosomal vesicle (Fig. 7a, boxed region, and b, g, k) tightly juxtaposed to the acroplaxome and to the F-actin hoops (marked by asterisks) of the apical ectoplasmic specialization in the peri-acrosomal (Fig. 7d, h), the sub-acrosomal (Fig. 7c, e, j), and the post-acrosomal (Fig. 7f, i) regions. Highly specialized structures such as the acroplaxome marginal ring (Fig. 7b, k, arrowheads and 7f, i, arrows), the manchette (Fig. 7b, i, k), the manchette perinuclear ring (Fig. 7b, f, i, k) as well as developing sperm tail (Fig. 7g) are clearly visible during the maturation phase. Cross sections of the flagellum show mitochondria gathered around the axoneme (Fig. 7l) to form the mitochondrial sheath in the sperm midpiece (Fig. 7m). In contrast, the principal piece of the sperm tail lacks mitochondria. However, fibrous material is present surrounding this tail region (Fig. 7n, arrows).

**Fig. 8:**
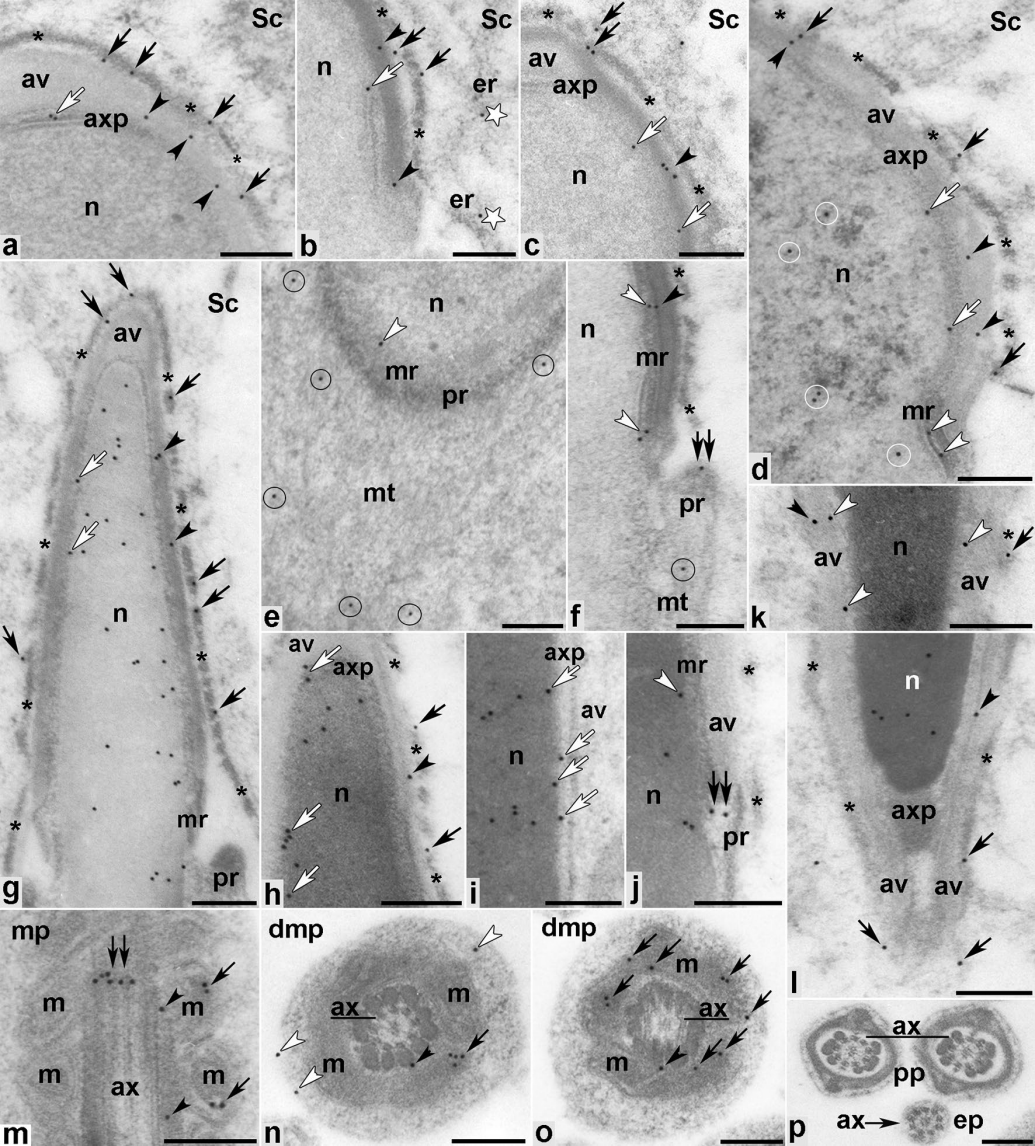
Immunogold localization of MVI in developing mouse spermatids during the maturation phase.* apx* acroplaxome,* av* acrosome vesicle,* ax* axoneme,* dmp* developing midpiece of the sperm tail,* ep* end piece of the sperm tail,* er* endoplasmic reticulum,* m* mitochondria,* mp* midpiece of the sperm tail,* mSpT* spermatid at the maturation phase,* mr* marginal ring of the acroplaxome,* mt* manchette,* n* nucleus,* pp* principal piece of the sperm tail,* pr* perinuclear ring of the manchette,* Sc* Sertoli cell. All other *indications* are explained in the text (see “Results”).* Bars *250 nm

As spermatid shaping progressed and the manchette developed just below the marginal ring of the acroplaxome, the MVI localization pattern remained similar to previous stages, predominantly detected in the F-actin hoops (marked by asterisks) surrounding the apical pole of the elongating nuclei (Fig. 8a–d, g, h, l, black arrows), the outer/inner acrosomal membranes (Fig. 8a–d, f, g, l, black arrowheads), and the acroplaxome interface (Fig. 8a–d, g–i, white arrows), including its marginal ring (Fig. 8d–f, j, k, white arrowheads). Some gold traces were also localized throughout the manchette flanking the elongating spermatid nucleus (Fig. 8e, f, circles) and in the perinuclear ring (Fig. 8f, j, double arrows). Interestingly, spermatid chromatin exhibited intense MVI immunoreactivity as condensation progressed (Fig. 8d, circles, and g–l). MVI was also found in the endoplasmic reticulum adjacent to the F-actin hoops within the Sertoli cytoplasm (Fig. 8b, white stars) and in developing flagellum (Fig. 8m–o). As expected, the axoneme was devoid of immunolabeling, but some gold traces were associated with the mitochondrial sheath in the midpiece of the flagellum (Fig. 8m–o, arrows) or localized at the axoneme periphery near the outer dense fibers (Fig. 8m–o, black arrowheads), where they occasionally form concentrated patches (Fig. 8m, double arrow). It was also noticeable that during the tail formation, MVI was detected in the tail residual cytoplasm (Fig. 8n, white arrowheads). MVI was absent in both the principal (Fig. 8p) and the end (Fig. 8p, arrow) pieces of the sperm tail. Control sections in which no MVI PAb was used were devoid of labeling (data not shown).

**Fig. 9:**
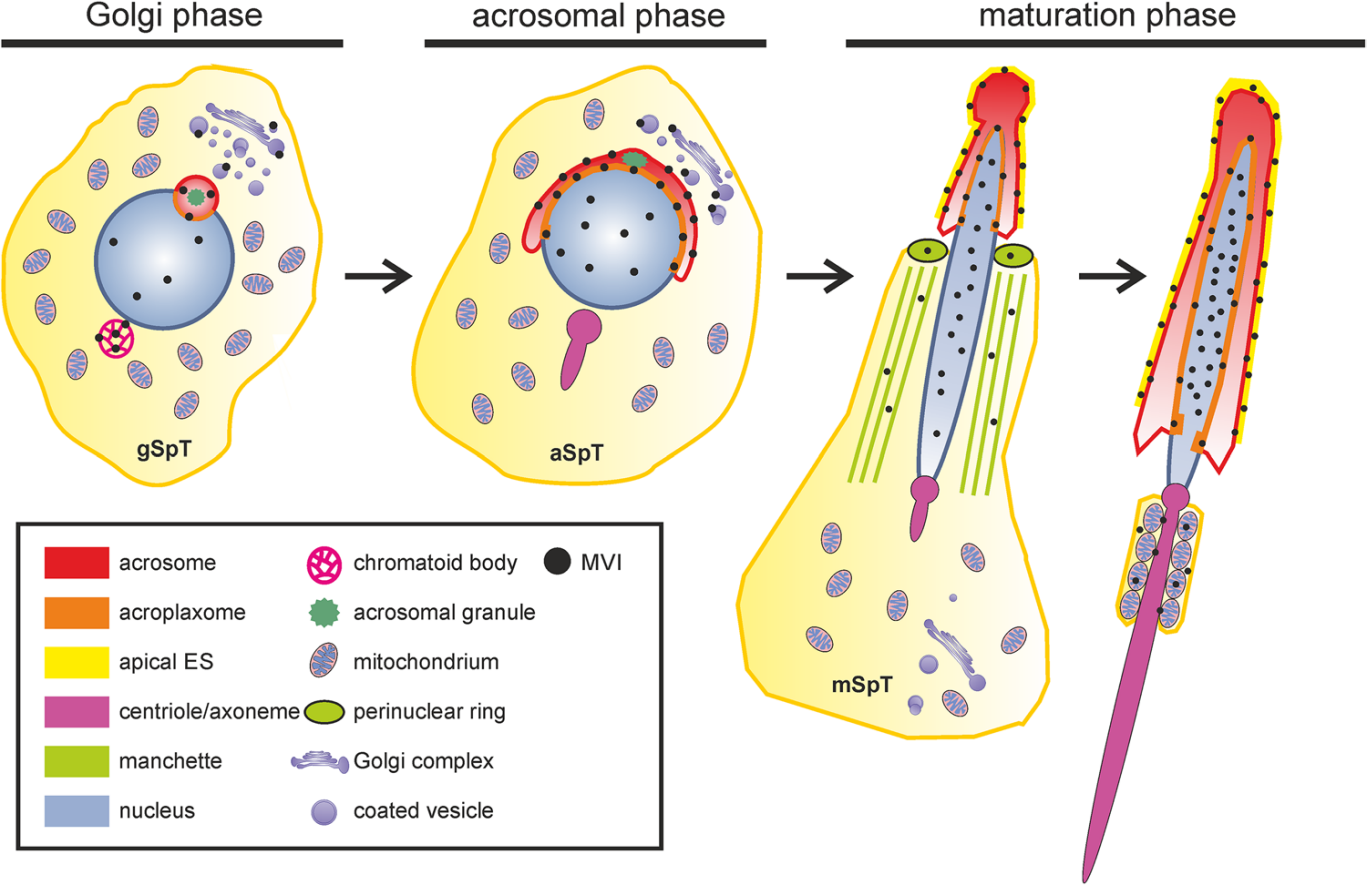
Schematic representation of MVI distribution (*black dots*) during mouse spermiogenesis.* aSpT* round spermatid at the acrosome phase,* gSpT* round spermatid at the Golgi phase,* mSpT* elongated spermatid at the maturation phase

Taken together, our immunocytochemical studies confirm MVI localization in highly specialized F-actin-containing structures present during mouse spermiogenesis, including the acrosome–acroplaxome–manchette complex and the apical ectoplasmic specializations surrounding the head region of the elongating spermatids. MVI was also prominently associated with the Golgi (including TGN), migrating chromatoid body, the nucleus, and mitochondrial sheath of the flagellum midpiece during the sperm tail development. A schematic summary of the obtained results is shown in Fig. 9.

## Discussion

Here, we present the first evidence that the unconventional actin-based motor protein, MVI, may be involved in some key events of spermiogenesis in mammals including: (1) acrosome biogenesis, (2) spermatid elongation, and (3) spermiation. We have also found that MVI-SI and MVI-NoI splice variants are the predominant isoforms expressed in mouse testes. Our findings are consistent with an earlier report showing that these MVI isoforms are expressed in rat testes (Buss et al. 2001). MVI immunolocalization shown here confirm that these isoforms are present in developing mouse spermatids and the Sertoli cells adjacent to them. Thus, we conclude that MVI with no LI splice variants are preferentially expressed in the seminiferous epithelia during spermiogenesis in mammals.

### MVI’s possible roles in acrosome biogenesis in mouse

Immunofluorescence and immunogold studies demonstrated the association of MVI with the anterior pole of developing spermatids, including the Golgi stacks and uncoated/clathrin-coated vesicles as well as the nascent, and then maturing acrosome–acroplaxome complex. These findings suggest that MVI may be involved in acrosomogenesis in two different ways. First, MVI association with vesicles is consistent with the idea that MVI plays a transport role in proacrosomal vesicle trafficking. Second, MVI association with the acrosome–acroplaxome complex suggests an anchoring role during the acrosome development.

Previous evidence indicates that proacrosomal vesicles utilize two different routes to reach their docking sites in the acroplaxome: a microtubule route, involving kinesin KIFC1 (Yang and Sperry 2003) and an F-actin route, involving MVa and Rab27a/b small GTPases that are known to play a general role in exocytosis (Kierszenbaum et al. 2003a, 2004). Moreover, it is believed that acrosome biogenesis involves both the anterograde vesicular transport from the TGN and the retrograde transport based on endocytic pathway (Ramalho-Santos et al. 2001 and see review by Berruti and Paiardi 2011). One of the best candidates to facilitate transport of vesicles derived from the Golgi and from the plasma membrane is MVI, which moves toward the minus end of actin filaments and is involved in both clathrin-mediated endocytosis and secretion (see review by Buss and Kendrick-Jones 2008). Interestingly, MVI-SI and MVI-NoI splice variants, the isoforms we detected in mouse testis, have previously been shown to play a role in transport of clathrin-coated as well as uncoated vesicles in different mammalian cell lines (Aschenbrenner et al. 2003; Dance et al. 2004; Naccache et al. 2006; Au et al. 2007; Chibalina et al. 2007; Puri 2009; Majewski et al. 2010; Bond et al. 2012; Tumbarello et al. 2012; Tomatis et al. 2013). Given that plus ends of actin filaments typically are positioned towards cell membranes (Cramer 1999), MVI acting as a minus-end-directed motor would move vesicles away from the Golgi surface to the center of the cell. MVI direct binding to multiple proteins involved in different steps along endocytic pathway (Chibalina et al. 2007; Spudich et al. 2007; Tumbarello et al. 2012 and see review by Tumbarello et al. 2013) and in Golgi complex (Sahlender et al. 2005) is consistent with this hypothesis. Additionally, a central role of MVI in exocytosis, including the Golgi ribbon formation and sorting of proteins into different cargo vesicles has been suggested. Specifically, MVI concentration in the TGN is thought to facilitate post-Golgi secretion, including vesicle formation and budding, and then fusion of secretory vesicles with the plasma membrane during the final stage of exocytosis (Buss et al. 1998; Warner et al. 2003; Au et al. 2007; Majewski et al. 2010; Bond et al. 2011; Tomatis et al. 2013). Thus, association of MVI with the Golgi apparatus, coated, and uncoated vesicles argue that MVI may be involved in the anterograde and/or the retrograde vesicular transport pathways during acrosome biogenesis in mouse.

Other hypotheses for MVI’s role are possible given previous work that support MVI ability to anchor various cargos to actin. For example, MVI-SI isoform controls neuroexocytosis in PC12 cells by tethering secretory granules to the actin network near the target plasma membrane (Tomatis et al. 2013). Given that MVa has been also implicated in neurosecretion (Tomatis et al. 2013 and references therein), these authors suggest synergistic roles for these two motor proteins, with MVI acting in recruitment and retention of secretory granules to the cortical actin network and MVa involved in their active transport toward the plasma membrane along actin filaments. Our results coupled with the previously documented role of MVa in acrosome formation could suggest a similar cooperation of these two molecular motors during acrosomogenesis in mouse.

In support of an anchoring role for MVI during acrosome development, this protein is strongly associated with the acrosome–acroplaxome complex, which is important for anchoring the acrosome to the nucleus. MVI is specifically located in developing acrosome–acroplaxome complex and its leading edges, including the outer/inner acrosomal membranes, the space between the acrosome membrane and the internal granule, the acroplaxome, and the acroplaxome marginal link. Such localizations suggest that MVI could help to mediate anchoring of the acrosome vesicle to the spermatid nucleus via the acroplaxome. The molecular basis of acrosome biogenesis, and particularly its attachment and spread over the nucleus, is poorly understood and to date only one protein, a transmembrane protein Dpy19l2, has been suggested to link the acrosome to the nuclear envelope (Pierre et al. 2012). However, the acroplaxome contains F-actin and actin-associated proteins such as cortactin and profilin-3 (Obermann et al. 2005; Kierszenbaum et al. 2008; Behnen et al. 2009), and potentially other ABPs (including motor proteins) which might modulate actin dynamics during acrosomogenesis and may be responsible for linking developing acrosome to the spermatid nucleus. A role for MVI in this linking is consistent with its crucial role in cell membrane tethering to cortical F-actin during development of the cochlear hair cells in the inner ear and the brush border cells in mammals (see reviews by Frank et al. 2004; Crawley et al. 2014).

We confirmed that actin accumulates in the acrosome–acroplaxome complex from the Golgi stage to the maturation stage. In contrast, MVI was initially localized in the nascent acrosomal sac, including its outer membrane, the inner membrane–acroplaxome site, and the interspace between the acrosomal membrane and the granule inside. MVI may be accumulated on the acrosomal membrane as a result of participating in transport of Golgi vesicles, before gaining access to the acroplaxome. At this time, we have no hypotheses about a potential role of MVI inside the acrosome vesicle.

An additional anchoring or structural role of MVI has been suggested in Golgi apparatus organization. In fibroblasts from *sv/sv* mice or cells depleted of MVI or its binding partner, optineurin, Golgi complexes were significantly altered in morphology and reduced in size compared to the wild-type cells (Warner et al. 2003; Sahlender et al. 2005; Majewski et al. 2011; Karolczak et al. 2015). In developing mouse spermatids at the Golgi stage, we found MVI associated with not only the TGN, but distributed throughout the Golgi apparatus from the *cis* to the *trans*-faces. This observation is consistent with potential structural role for Golgi-localized MVI during mouse spermiogenesis. However, we have not been able to confirm so far that optineurin is a MVI binding partner in developing mouse spermatids (data not shown).

### MVI may play a role in nuclear shaping during the maturation stage

The association of MVI with the actin structures that mediate sperm nuclear shaping suggests an important role during the elongation stage. Nuclear shaping is a critical event during sperm development involving the condensation of the future sperm nucleus from a spherical configuration into an elongated structure. Two structural components of maturing spermatids are critical in this process—the acroplaxome marginal ring and the manchette, both containing actin filaments. These structures together with F-actin hoops, the main cytoskeletal element of the Sertoli cell apical ectoplasmic specializations, seem to stabilize the spermatid head as it is undergoing elongation and to mediate the sperm–Sertoli cell association during spermiation (see reviews by Kierszenbaum and Tres 2004; Kierszenbaum et al. 2011; Sun et al. 2011; Qian et al. 2014; Xiao et al. 2014). The acroplaxome marginal ring, the manchette, and actin dynamics within them seem to be important in modulation of clutching forces that mediate spermatid head elongation. On the other hand, the Sertoli cell actin hoops surrounding the elongating spermatid head also are important for nuclear shaping. Numerous testis-specific ABPs that regulate actin assembly/disassembly have been identified in the acroplaxome–manchette complex and the apical tubulobulbar–Sertoli cell ectoplasmic specialization complexes in mammals, including profilin-3, gelsolin, Eps8, Arp2/3, drebrin E, Rai14, and palladin (Braun et al. 2002; Guttman et al. 2002; Lie et al. 2009, 2010; Li et al. 2011; Qian et al. 2013a, b). The components and mechanism of action of these actin-regulating protein complexes are poorly understood. We postulate that a key element of these protein complexes may be MVI. This protein is continuously associated with the acroplaxome–manchette complex from the early acrosome stage to the late maturation stage, and with the actin hoops surrounding the elongating spermatid/sperm head. Phosphorylated cortactin is also present in both the acroplaxome and the Sertoli cell hoops whereas non-phosphorylated cortactin predominates in the manchette (Kierszenbaum et al. 2008). Phosphorylated cortactin interacts with the actin nucleator, Arp2/3 complex, that allows actin remodeling from bundled to branched configuration (Weaver et al. 2001; Young et al. 2012 and see reviews by Cheng and Mruk 2011; Qian et al. 2014). Our previous studies showed that during *Drosophila* spermatid individualization MVI colocalizes with cortactin and Arp2/3 in the front meshwork of actin cones, and that lack of functional MVI results in impaired distribution of these ABPs (Rogat and Miller 2002; Noguchi et al. 2008; Isaji et al. 2011). One model for the MVI role in actin cone structure stabilization is anchoring specific cargos to the front meshwork. We postulate that MVI may play a similar role in developing mouse spermatids by anchoring actin regulators that are responsible for mediating the actin dynamics required for forces involved in nuclear shaping.

### Possible additional roles for MVI during sperm maturation

MVI is associated with the chromatoid body at the Golgi stage, a cloud-like structure packed with RNA and many of RNA-binding and RNA-processing proteins (see review by Meikar et al. 2011). It also progressively accumulates in the spermatid nucleus from the Golgi stage to the maturation stage. In addition, MVI was present in condensing spermatid nucleus. Previous work showed that MVI colocalizes with RNA polymerase II and newly synthesized mRNA transcripts (Jung et al. 2006; Vreugde et al. 2006) and may play a role in nucleo-cytoplasmic trafficking (Majewski et al. 2010). Thus, additional roles for MVI are possible in nuclear processes. In addition, association with the mitochondrial sheath may suggest a role in formation of this structure. Much less is known about binding proteins that might mediate MVI association with and specific functional roles for MVI in such structures and compartments. Finally, actin structures are important for movement of spermatids across the seminiferous epithelium and for spermiation, and MVI association with those structures may also be important. Additional studies are required to define the processes in which MVI plays a role and its precise function(s) throughout sperm differentiation.

## Conclusion

Based on the strong and prominent association of MVI with a number of structures important for key steps of spermiogenesis, we suggest dual roles for MVI in this process. First, MVI may play a transport role during formation the Golgi–acrosome–acroplaxome–manchette complex. Second, MVI may play an anchoring role in maintaining the morphology of the Golgi complex and may help tether actin regulators to several unique F-actin-containing structures involved in acrosomal attachment to the nucleus, acrosomal cap formation, and nuclear shaping: the acroplaxome, the manchette and the apical ectoplasmic specializations of Sertoli cells. In addition, MVI along with other ABPs may play additional roles in a variety of other processes since MVI was also observed to be associated with chromatoid bodies, mitochondrial sheath of the tail midpiece, and the condensing nucleus. Future studies using MVI mutant males and identification of the testis-specific MVI binding partner/s are needed to verify these hypotheses.

## Abbreviations

ABP/s: Actin-binding/regulating protein(s)
AF/s: Actin filament(s)
F-actin: Filamentous actin
JLA20 MAb: Monoclonal antibody against actin
LI: Large insert in MVI tail region
MVa: Myosin Va
MVI: Myosin VI
MVI PAb: Polyclonal antibody against MVI
NoI MVI: Isoform with no inserts in the tail domain
SI: Small insert in MVI tail region
*sv*/*sv* mice: *Snell’s waltzer* mutants that lack MVI
TGN: *trans*-Golgi network
